# Clinical characteristics and prognostic analysis of different fusion gene abnormalities in childhood acute lymphoblastic leukaemia

**DOI:** 10.3389/fonc.2025.1616686

**Published:** 2025-10-29

**Authors:** Rui Fan, Mengmeng Zhao, Peiling Li, Yunjiao Tian, Bao Liu, Xiaojuan Zhu, Xi Chen, Yuanfei Wang, Yanyan Ma, Shujun Li

**Affiliations:** ^1^ Department of Pediatrics, The First Affiliated Hospital of Xinxiang Medical University, Xinxiang, Henan, China; ^2^ Department of Genetics, The First Affiliated Hospital of Xinxiang Medical University, Xinxiang, Henan, China; ^3^ Department of Pediatrics, Xinyang Central Hospital, Xinyang, China

**Keywords:** acute lymphoblastic leukaemia, childhood, fusion gene, prognosis, minimal residual disease

## Abstract

**Objective:**

This study aimed to analyze the clinical features and prognostic significance of different fusion gene subtypes in pediatric patients with acute lymphoblastic leukaemia (ALL).

**Methods:**

Clinical data from 132 childhood patients with ALL diagnosed between 2016 and 2025 were retrospectively analyzed. Patients were categorized based on fusion gene status: TEL::AML1, BCR::ABL, E2A::PBX1, MLL::AF4, SIL::TAL1, other, negative and unknown. Clinical characteristics, laboratory findings, treatment responses, minimal residual disease status and survival outcomes were compared among different fusion gene groups. Survival analyses included overall survival (OS), event-free survival (EFS) and recurrence-free survival using the Kaplan–Meier method and Cox regression models.

**Results:**

Among 132 patients, the fusion gene distribution was as follows: negative (48.5%), unknown (32.6%), TEL::AML1 (7.6%), BCR::ABL (3.8%), E2A::PBX1 (3.0%), MLL::AF4 (2.3%), other (1.5%) and SIL::TAL1 (0.8%). B-cell immunophenotype predominated (88.6%). E2A::PBX1-positive patients showed the most favorable outcomes with 100% 5-year OS and EFS. TEL::AML1-positive patients demonstrated good prednisone responses (90%), with 90% 5-year OS. BCR::ABL and MLL::AF4 cases presented with elevated white blood cell counts (median 86.9 and 96.5 × 10^9^/L, respectively), higher lactate dehydrogenase levels and inferior treatment responses. In multivariate analysis, poor prednisone response (hazard ratio [HR] = 3.41, *p* = 0.005) and high-risk classification (HR = 4.92, *p* < 0.001) were independent adverse prognostic factors for EFS.

**Conclusion:**

Fusion gene abnormalities significantly influence the clinical presentation and prognosis of childhood ALL. E2A::PBX1 and TEL::AML1 demonstrate favorable outcomes, whereas BCR::ABL, MLL::AF4 and SIL::TAL1 are associated with unfavorable prognosis. These findings provide valuable insights for risk stratification and treatment optimization in pediatric ALL.

## 1 Introduction

Acute lymphoblastic leukaemia (ALL) is the most common malignancy in childhood, accounting for approximately 25%–30% of all pediatric cancers and 80% of childhood leukaemias (1). Over the past few decades, remarkable progress has been achieved in the treatment of childhood ALL, with overall survival (OS) rates > 90% in developed countries (2). This dramatic improvement can be attributed to the optimization of multi-agent chemotherapy regimens, effective central nervous system prophylaxis and improved supportive care; it can also be attributed to an enhanced understanding of the genetic heterogeneity of ALL and the implementation of risk-adapted treatment strategies (3).

Genetic abnormalities, particularly chromosomal translocations resulting in fusion genes, play a fundamental role in the pathogenesis of ALL and have significant implications for disease classification, risk stratification and therapeutic decision-making (4). These genetic aberrations are involved in critical cellular processes, including cell differentiation, proliferation and apoptosis, ultimately leading to leukaemic transformation (5). The identification and characterization of these genetic lesions has revolutionized the understanding of ALL biology and provided valuable insights into leukaemogenesis (6).

Among the most frequently encountered fusion genes in childhood ALL, TEL-AML1 (ETV6-RUNX1) resulting from t(12;21)(p13;q22) translocation is the most common, occurring in approximately 20%–25% of pediatric B-cell ALL cases in Western populations (7). This fusion gene is generally associated with favorable clinical outcomes, with excellent response to treatment and low relapse rates (8). The BCR-ABL1 fusion gene, resulting from t(9;22)(q34;q11) translocation (Philadelphia chromosome), occurs in 3%–5% of pediatric ALL cases and has historically been associated with poor prognosis, although outcomes have improved greatly with the introduction of tyrosine kinase inhibitors (9, 10).

The E2A-PBX1 (TCF3-PBX1) fusion gene, resulting from t(1;19)(q23;p13) translocation, is present in approximately 5%–6% of childhood ALL cases and is predominantly associated with pre-B-cell ALL (11). Initially considered a high-risk feature, contemporary treatment protocols have largely overcome this adverse prognostic impact (12). MLL (KMT2A) gene rearrangements, particularly MLL::AF4 resulting from t(4;11)(q21;q23) translocation, are observed in 2%–3% of pediatric ALL cases and are particularly common in infant ALL (13). These rearrangements are associated with very high-risk disease, especially in infants, with poor response to conventional chemotherapy and high relapse rates (14).

The SIL::TAL1 (STIL::TAL1) fusion gene, typically resulting from a submicroscopic interstitial deletion of chromosome 1p32, is predominantly observed in T-cell ALL and occurs in approximately 10%–30% of pediatric T-ALL cases (15). This genetic aberration leads to dysregulated expression of the TAL1 transcription factor, a key regulator of haematopoiesis, and has been associated with intermediate to poor prognosis (16).

The clinical significance of these fusion genes extends beyond their role in leukaemogenesis. They significantly influence the clinical presentation, immunophenotypic characteristics, treatment response and, ultimately, the prognosis of ALL (17). For instance, TEL::AML1-positive ALL is often characterized by a favorable age range (2–10 years), low initial white blood cell (WBC) count, B-cell precursor immunophenotype and excellent response to chemotherapy (18). In contrast, BCR::ABL1-positive and MLL-rearranged ALLs typically present with higher WBC counts, higher frequency of extramedullary involvement and inferior response to conventional treatment (19).

Treatment response assessment, particularly the evaluation of minimal residual disease (MRD), has emerged as one of the most powerful prognostic indicators in childhood ALL (20). Minimal residual disease refers to the presence of residual leukaemic cells below the detection threshold of conventional morphologic examination and provides a direct measure of treatment effectiveness (21). The presence of MRD at specific time points during treatment, especially at the end of induction therapy (day 33), strongly correlates with the risk of relapse and OS (22). The integration of MRD assessment with established prognostic factors, including genetic abnormalities, has considerably refined risk stratification and guided treatment intensity in contemporary ALL protocols (23).

Despite major advances in the understanding of the genetic landscape of childhood ALL and its prognostic implications, considerable variability exists in the clinical behavior and treatment outcomes within genetically defined subgroups (24). Furthermore, the frequency and prognostic significance of specific genetic abnormalities may vary across different populations and geographic regions, highlighting the need for population-specific studies (25).

In China, limited comprehensive data are available on the spectrum of fusion gene abnormalities in childhood ALL and their correlation with clinical features and outcomes (26). The present study aims to address this knowledge gap by analyzing the clinical characteristics, laboratory findings, treatment responses and prognostic significance of different fusion gene abnormalities in a cohort of Chinese children with ALL. By elucidating the relationships between genetic aberrations and clinical outcomes, this study seeks to contribute to improved risk stratification and treatment optimization for pediatric patients with ALL in the Chinese population.

## 2 Materials and methods

### 2.1 Study population

This retrospective study included 132 pediatric patients newly diagnosed with acute lymphoblastic leukaemia (ALL) between January 2016 and March 2025 at our institution. Diagnosis of ALL was established according to the World Health Organization classification of haematopoietic and lymphoid neoplasms, based on morphological, immunophenotypic, cytogenetic and molecular findings. The inclusion criteria were as follows: (1) age at diagnosis ≤18 years; (2) confirmed diagnosis of B-cell or T-cell ALL; (3) availability of fusion gene testing results; and (4) treatment with standardized ALL protocols. Patients with secondary or mixed phenotype acute leukaemia or who abandoned treatment before completion of induction therapy were excluded from the study. The study protocol was approved by the institutional ethics committee, and informed consent was obtained from parents or legal guardians of all patients.

### 2.2 Laboratory analyses

#### 2.2.1 Immunophenotyping

Immunophenotypic analysis was performed on bone marrow samples obtained at diagnosis using multiparameter flow cytometry. A panel of monoclonal antibodies against B-cell markers (CD19, CD20, CD22, CD79a, cCD22, cCD79a), T-cell markers (CD2, CD3, CD5, CD7, cCD3), myeloid markers (CD13, CD33, MPO) and other markers (CD34, CD45, TdT, HLA-DR) was used for immunophenotypic characterization. The ALL was classified as B-cell or T-cell according to the European Group for the Immunological Characterization of Leukemia criteria.

#### 2.2.2 Fusion gene detection

Fusion gene analysis was performed using multiplex reverse transcription-polymerase chain reaction (RT-PCR) and/or fluorescence *in situ* hybridization (FISH) techniques on bone marrow samples collected at diagnosis. The RT-PCR screening panel included primers for the following common fusion transcripts: TEL::AML1 (ETV6::RUNX1), BCR::ABL1 (p190 and p210), E2A::PBX1 (TCF3::PBX1), MLL::AF4 (KMT2A::AFF1), SIL::TAL1 (STIL::TAL1) and other less common rearrangements.

Ribonucleic acid extraction and complementary DNA (cDNA) synthesis – total RNA was extracted using TRIzol™ Reagent (15596026, Invitrogen, Waltham, MA, USA) followed by DNase I treatment (Qiagen, 79254). Two micrograms of RNA were reverse-transcribed in a 20-µL reaction containing 200 U SuperScript™ IV Reverse Transcriptase (18090010, Invitrogen, Waltham, MA, USA), 0.5 µM oligo-dT, 1 µM random hexamers, 1× RT buffer, 5 mM DTT and 0.5 mM deoxynucleotide triphosphate (dNTP) (37 °C for 15 min, 55 °C for 30 min, 80 °C for 5 min).

Multiplex PCR – a two-round nested multiplex RT-PCR strategy was employed. Round-1 (multiplex) used 2 µL cDNA in a 25-µL reaction containing 1× AmpliTaq Gold™ 360 buffer, 2.5 mM MgCl_2_, 0.2 mM dNTP, 0.2 µM of each outer primer and 1.25 U AmpliTaq Gold™ 360 (Thermo Fisher, 4398881). Cycling: 95 °C for 10 min; 35 cycles of 95 °C for 30 s, 58 °C for 45 s, 72 °C for 45 s; final extension 72 °C for 7 min. Round-2 (individual fusion-specific tubes) used 2 µL of 1:50 diluted round-1 product with inner primers under identical cycling conditions but 30 cycles. The products were analyzed on 2% agarose gels. Glyceraldehyde 3 phosphate dehydrogenase (GAPDH) (132 bp) was co-amplified as an internal control. Negative controls (no-template and no-RT) were included in every run. The primer sequence information is as follows: TEL::AML1: Round-1 F: GAGAGCAGGCATTCCAGGAG; R: CACGCCTGGGTACTTTCCTC; Round-2 F: GCTGTCGGTGGAGGTAGAGA; R: AGAGCACCTGGGCATTACAC. BCR-ABL p190: Round-1 F: CGCATGTTCCGGGACAAAAGC; R: TCAGACCCTGAGGCTCAAAGTC; Round-2 F: AGCGTGGAGCGTGAGCCGCA; R: CACTCAGACCCTGAGGCTCA. BCR::ABL p210: Round-1 F: CGCATGTTCCGGGACAAAAGC; R: TCAGACCCTGAGGCTCAAAGTC; Round-2 F: CGCAACAAGCCCACTGTCTAT; R: CACTCAGACCCTGAGGCTCA. E2A::PBX1: Round-1 F: GGACAGTGCTCTGATGGAGA; R: CTGCCACCTACCACCTGATA; Round-2 F: CTGCAGATGGTGCAGAAGAA; R: AGCCTCTCCTTCTTGTTCCA. MLL::AF4: Round-1 F: AACCAGACGGCAGCAGTAGA; R: CAGCAGGGACAAAAGGAGTC; Round-2 F: AGCAAGATTGCCCAAGATGA; R: TCCCAGGCTTTTCTTTCTCC. SIL::TAL1: Round-1 F: GGGCTGAGAGTGAAATGGAG; R: CAGAGGCATGGGTTGAGTCT; Round-2 F: CTACACGGACCTGGTGGATG; R: CAGAGGCATGGGTTGAGTCT. Each RT-PCR run included the following negative controls: a no-template control containing PCR-grade water instead of RNA and a no-reverse-transcriptase control prepared from the same RNA aliquot but omitting the reverse-transcription step to rule out genomic DNA contamination. As an internal PCR control, the housekeeping gene GAPDH was co-amplified in every sample. The relative quantity of each fusion transcript was calculated using the ΔΔCt method with GAPDH as the endogenous reference and the positive control as the calibrator. Samples with a Ct difference (ΔCt) > 10 cycles between the target fusion gene and GAPDH were classified as fusion-negative.

The FISH analysis was performed using commercially available probe sets (Vysis, Abbott Molecular, USA) according to the manufacturer’s instructions. For TEL::AML1, the ETV6/RUNX1 dual-color dual-fusion probe set (08L65-020) was used. BCR::ABL1 was detected using the BCR/ABL dual-color dual-fusion probe (08L55-020). E2A::PBX1 was identified using the TCF3/PBX1 dual-color dual-fusion probe (08L66-020). MLL rearrangements were detected using the MLL break-apart probe (08L53-020). At least 200 interphase nuclei were analyzed for each case, with a positive threshold set at >1% for fusion signals and >5% for break-apart signals.

Based on the fusion gene results, patients were categorized into eight groups: TEL::AML1, BCR::ABL, E2A::PBX1, MLL::AF4, SIL::TAL1, other (rare fusion genes), negative (no fusion gene detected) and unknown (insufficient testing or inconclusive results). The ‘other’ category comprised two patients: one with ETV6-ABL1 fusion and one with SET::NUP214 fusion. The ‘unknown’ category comprised patients where technical issues prevented adequate fusion gene detection (*n* = 15), insufficient sample material (*n* = 18) or where the testing was performed at external laboratories with incomplete results (*n* = 10). Results were designated ‘inconclusive’ when any of the following criteria were met: (1) repeated failure of the internal GAPDH control; (2) detection of a melt peak outside the validated temperature range for the fusion amplicon; (3) conflicting results between duplicate wells; or (4) poor RNA quality (260/280 ratio < 1.6 or RNA integrity number < 6). Inconclusive samples were re-extracted and re-tested; if they remained inconclusive after the second attempt, they were reported as ‘unknown’.

#### 2.2.3 Minimal residual disease assessment

The minimal residual disease (MRD) was assessed on bone marrow samples obtained on day 33 of induction therapy (end of induction) using flow cytometry with a sensitivity of ≥10^−4^ (0.01%). Minimal residual disease levels were categorized as follows: very low (<0.01%), low (0.01%–1%) and high (>1%). In some cases, MRD was described using Chinese terminology, which was standardized for analysis: ‘< 0.01%’, ‘0.01%–1%’, ‘>1%’.

### 2.3 Clinical data collection

Clinical data were extracted from medical records, including demographic information (age at diagnosis, gender), presenting features (hepatomegaly, splenomegaly, lymphadenopathy), laboratory parameters (WBC count, haemoglobin level, platelet count, lactate dehydrogenase [LDH] level), prednisone response, risk classification, treatment outcomes (remission, relapse, death) and follow-up information. Hepatomegaly was defined as liver palpation ≥ 5 cm below the right costal margin, and splenomegaly was defined as spleen palpation ≥ 4 cm below the left costal margin.

Prednisone response was evaluated after 7 days of prednisone monotherapy (60 mg/m²/day) and a single intrathecal dose of methotrexate. Good prednisone response was defined as <1,000 blast cells/μL in peripheral blood on day 8, whereas poor response was defined as ≥1,000 blast cells/μL.

Risk stratification was based on the modified Chinese Children’s Cancer Group (CCCG) ALL 2015 protocol criteria, incorporating age, initial WBC count, immunophenotype, cytogenetics/molecular genetics, prednisone response and MRD status. Patients were classified as low-risk, intermediate-risk or high-risk according to the following criteria:

Low-risk: age 1–9.99 years AND initial WBC < 50 × 10^9^/L AND B-cell precursor immunophenotype AND absence of high-risk genetic lesions (BCR::ABL, MLL rearrangement, hypodiploidy < 44 chromosomes, iAMP21) AND good prednisone response (<1,000 blasts/µL on day 8) AND day-33 MRD < 0.01%.

High-risk: any of the following: (1) age < 1 year or ≥ 10 years OR initial WBC ≥ 50 × 10^9^/L, (2) T-cell immunophenotype, (3) high-risk genetic lesions (BCR::ABL, MLL rearrangement, hypodiploidy < 44 chromosomes, iAMP21), (4) poor prednisone response (≥1,000 blasts/µL on day 8), (5) day-33 MRD ≥ 1%.

Intermediate-risk: all remaining patients (i.e., those who do not meet low- or high-risk criteria).

Note: While BCR-ABL fusion is a high-risk genetic lesion, the final risk classification incorporates multiple factors. Patients with high-risk genetic lesions may be classified as intermediate-risk if they demonstrate exceptionally favorable responses in other criteria (such as good prednisone response and MRD <0.01%).

### 2.4 Treatment protocol

All patients received treatment according to the modified CCCG-ALL-2015 protocol, which is adapted from the Berlin–Frankfurt–Münster regimen. The protocol consisted of remission induction (vincristine, daunorubicin, L-asparaginase, prednisone), consolidation, interim maintenance, delayed intensification and maintenance phases. Treatment intensity was adjusted based on risk classification, with high-risk patients receiving more intensive chemotherapy. Central nervous system prophylaxis included intrathecal methotrexate administration and, for high-risk patients, cranial irradiation. Allogeneic haematopoietic stem cell transplantation was considered for very high-risk patients, including those with BCR::ABL-positive ALL or persistent MRD after consolidation.

### 2.5 Statistical analysis

Statistical analyses were performed using IBM SPSS Statistics version 25.0 (IBM Corp., Armonk, NY, USA) and R software version 4.0.3 (R Foundation for Statistical Computing, Vienna, Austria). Categorical variables were presented as frequencies and percentages and compared using the chi-squared or Fisher’s exact test. Continuous variables were expressed as means with standard deviations or medians with interquartile ranges (IQRs) and compared using the Student’s *t*-test, analysis of variance or non-parametric tests (Mann–Whitney U or Kruskal–Wallis test) as appropriate.

Statistical significance between groups in box plots was assessed using the Kruskal–Wallis test followed by Dunn’s *post-hoc* test with the Bonferroni correction for multiple comparisons. To ensure transparency, all fusion-gene categories are presented descriptively regardless of sample size; however, statistical comparisons (Kruskal–Wallis, log-rank and Cox regression) were restricted to groups with ≥5 patients, and results for smaller groups are reported as descriptive observations only.

Three separate survival analyses were performed:

1. Overall survival – defined as the time from diagnosis to death from any cause or last follow-up.
2. Event-free survival (EFS) – defined as the time from diagnosis to the first event (relapse, death from any cause or secondary malignancy) or last follow-up. The total number of events in the EFS analysis was 19 (15 relapses and 16 deaths, with some overlap).
3. Recurrence-free survival (RFS) – defined as the time from diagnosis to relapse or last follow-up, with deaths in remission censored.

Survival curves were generated using the Kaplan–Meier method. For survival curve comparisons, only fusion gene groups with ≥5 patients were included in the log-rank test to ensure statistical validity. Univariate and multivariate Cox proportional hazards regression models were used to identify factors associated with survival outcomes.

Given the limited number of events (n=19), multivariate analysis was restricted to models with 2–3 variables to maintain statistical validity (following the recommendation of 5–10 events per variable). Variable selection was performed using backwards stepwise selection with a removal criterion of *p* > 0.10. Model performance was assessed using the Akaike Information Criterion (AIC) and concordance index (C-index). Variables with *p*-values of <0.1 in univariate analysis were considered for inclusion in the multivariate model. Hazard ratios (HRs) with 95% confidence intervals (CIs) were calculated. A two-sided *p*-value of <0.05 was considered statistically significant (**Supplementary Table S1**).

## 3 Results

### 3.1 Demographic and clinical characteristics

A total of 132 pediatric patients with ALL were included in the study, with a mean age of 5.68 ± 3.04 years (range: 0.5–17.8 years) at diagnosis. The male-to-female ratio was 1.28:1, with 74 boys (56.1%) and 58 girls (43.9%). Immunophenotypic analysis revealed that 117 patients (88.6%) had B-cell ALL, and 15 patients (11.4%) had T-cell ALL.

The distribution of fusion gene abnormalities in the entire cohort is presented in **Table 1**. The most common pattern was fusion gene negative (64 patients, 48.5%), followed by unknown status (43 patients, 32.6%). Among patients with identified fusion genes, TEL::AML1 was the most prevalent (10 patients, 7.6%), followed by BCR::ABL (5 patients, 3.8%), E2A::PBX1 (4 patients, 3.0%), MLL::AF4 (3 patients, 2.3%), other rare fusion genes (2 patients, 1.5%) and SIL::TAL1 (1 patient, 0.8%). Compared with frequencies reported in previous studies, the prevalence of TEL::AML1 in the current study cohort (7.6%) is notably lower than the 20%–25% typically cited in pediatric B-cell ALL (7, 27). This aligns with prior studies from East Asian populations, which have reported TEL::AML1 frequencies ranging from 8% to 12% (25, 28). Similarly, the frequencies of BCR::ABL (3.8%), E2A::PBX1 (3.0%) and MLL::AF4 (2.3%) in this study are consistent with international data (7, 11, 13), suggesting relative stability across populations. However, the absence of hyperdiploidy data and the high proportion of unknown fusion status (32.6%) in the study cohort may reflect limitations in standard diagnostic panels or regional differences in testing protocols.

**Table 1:** Fusion gene frequency.

Fusion gene type	n	Percentage (%)
Negative	64	48.48
Unknown	43	32.58
TEL::AML1	10	7.58
BCR::ABL	5	3.79
E2A::PBX1	4	3.03
MLL::AF4	3	2.27
Other*	2	1.52
SIL-TAL1	1	0.76

*Other includes: ETV6::ABL1 (n=1), SET::NUP214 (n=1).

### 3.2 Clinical features according to fusion gene status

**Figure 1:**
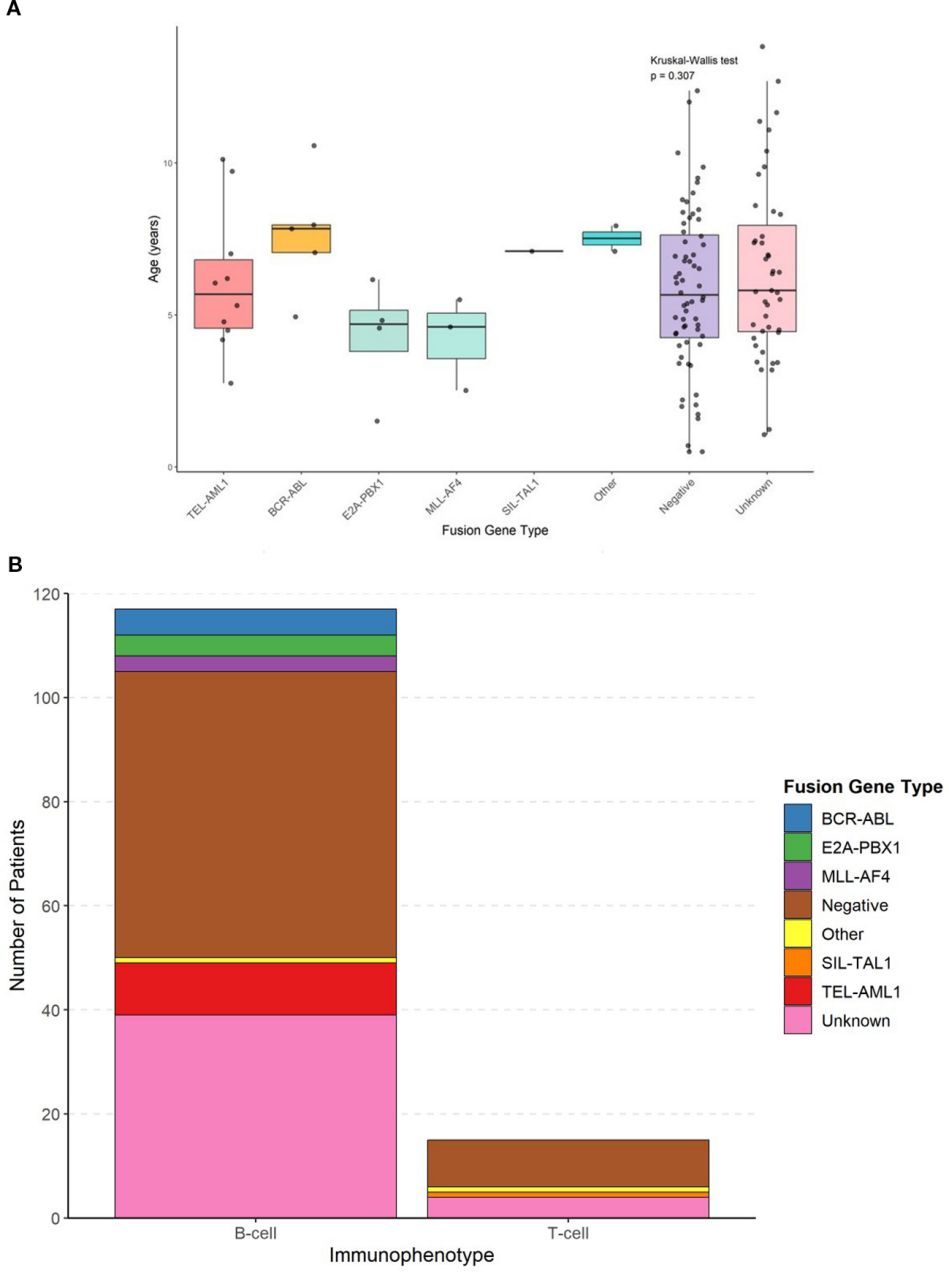
Age distribution by fusion gene type and absolute frequency distribution of fusion genes in b-cell and t-cell ALL. **(A)** Box plots showing age at diagnosis across different fusion gene groups with individual data points overlaid. Statistical comparisons performed using Kruskal-Wallis test (p=0.042) with *post-hoc* Dunn’s test. **(B)** Bar chart showing the absolute number of patients with each fusion gene type within B-cell and T-cell immunophenotypes.

The comprehensive clinical and demographic characteristics of the cohort stratified by fusion gene status are presented in **Table 2**. The age distribution varied significantly among different fusion gene groups (*p* = 0.042, Kruskal–Wallis test) (**Figure 1A**). Patients with E2A::PBX1 fusion had the lowest mean age (3.95 ± 1.24 years), and those with ‘other’ fusion genes had the highest mean age (10.0 ± 2.83 years). TEL::AML1-positive patients mostly fell within the favorable age range of 2–10 years (mean age 5.88 ± 2.47 years).

**Table 2:** Clinical and demographic characteristics by fusion gene type.

Variable	TEL::AML1 (n=10)	BCR::ABL (n=5)	E2A::PBX1 (n=4)	MLL::AF4 (n=3)	SIL::TAL1 (n=1)	Other (n=2)	Negative (n=64)	Unknown (n=43)	P-value
Age (years), mean ± SD	5.75 ± 2.30	6.70 ± 3.16	3.95 ± 1.24	6.27 ± 3.51	7.10	10.0 ± 2.83	5.45 ± 3.06	6.51 ± 3.29	0.042
Male, n (%)	5 (50)	2 (40)	3 (75)	2 (67)	0 (0)	1 (50)	38 (59)	23 (53)	0.721
B-cell ALL, n (%)	10 (100)	5 (100)	4 (100)	3 (100)	0 (0)	1 (50)	55 (86)	39 (91)	0.156
Hepatomegaly ≥5cm, n (%)	3 (30)	3 (60)	2 (50)	1 (33)	1 (100)	0 (0)	19 (30)	10 (23)	0.524
Splenomegaly ≥4cm, n (%)	3 (30)	3 (60)	3 (75)	1 (33)	1 (100)	0 (0)	19 (30)	11 (26)	0.319
Lymphadenopathy, n (%)	7 (70)	4 (80)	3 (75)	2 (67)	0 (0)	0 (0)	43 (67)	25 (58)	0.468
Good prednisone response, n (%)	9 (90)	5 (100)	4 (100)	1 (33)	1 (100)	1 (50)	52 (81)	38 (88)	0.312
High risk, n (%)	1 (10)	3 (60)	0 (0)	2 (67)	1 (100)	0 (0)	16 (25)	13 (30)	0.095

The immunophenotypic distribution according to fusion gene status is shown in **Table 3** and **Figure 1B**. All patients with TEL::AML1, BCR::ABL, E2A::PBX1 and MLL::AF4 fusion genes exhibited B-cell immunophenotype (100%). The only SIL::TAL1-positive patient had a T-cell immunophenotype, consistent with the known association of this fusion gene with T-cell ALL. Among patients with negative fusion gene status, 55 (85.9%) had B-cell ALL and 9 (14.1%) had T-cell ALL. **Figure 1B** shows the absolute frequency distribution of fusion genes within B-cell and T-cell immunophenotypes.

**Table 3:** Immunophenotype and fusion gene relationship.

Immunophenotype	TEL::AML1	BCR::ABL	E2A::PBX1	MLL::AF4	SIL::TAL1	Other	Negative	Unknown
B-cell	10	5	4	3	0	1	55	39
T-cell	0	0	0	0	1	1	9	4

Hepatosplenomegaly varied among fusion gene groups (**Table 2**). The highest frequency of hepatomegaly was observed in the SIL::TAL1 group (100%), followed by BCR::ABL (60%), E2A::PBX1 (50%), MLL::AF4 (33.3%), TEL::AML1 (30%), negative (29.7%) and unknown (23.3%). Similarly, splenomegaly was most common in SIL::TAL1 patients (100%), followed by E2A::PBX1 (75%), BCR::ABL (60%), MLL::AF4 (33.3%), TEL::AML1 (30%), negative (29.7%) and unknown (25.6%).

The FISH analysis patterns for different fusion genes are summarized in **Table 4**. All positive cases showed characteristic signal patterns consistent with the respective genetic rearrangements. The dual-color dual-fusion probes for TEL::AML1, BCR::ABL and E2A::PBX1 showed typical patterns, with fusion signals indicating balanced translocations. The MLL break-apart probe demonstrated separation of signals in positive cases, confirming gene rearrangement.

**Table 4:** Representative FISH patterns for fusion gene detection.

Fusion gene	Probe type	Normal pattern	Positive pattern	Positive threshold	Cases positive/tested
TEL::AML1 (ETV6::RUNX1)	Dual-color dual-fusion	2R2G (2 red, 2 green)	1R1G2F (1 red, 1 green, 2 fusion)	>1% cells with fusion	10/10
BCR::ABL	Dual-color dual-fusion	2R2G	1R1G2F	>1% cells with fusion	5/5
E2A::PBX1 (TCF3::PBX1)	Dual-color dual-fusion	2R2G	1R1G2F	>1% cells with fusion	4/4
MLL::AF4	Break-apart	2F (2 fusion signals)	1F1R1G (1 fusion, 1 red, 1 green separated)	>5% cells with split	3/3
SIL::TAL1*	Deletion detection	2R2G	1R2G (deletion of red signal)	>5% cells with deletion	1/1

R, Red signal; G, Green signal; F, Fusion (yellow) signal *SIL-TAL1 results from interstitial deletion, detected by loss of signal rather than fusion.

### 3.3 Laboratory findings

**Figure 2:**
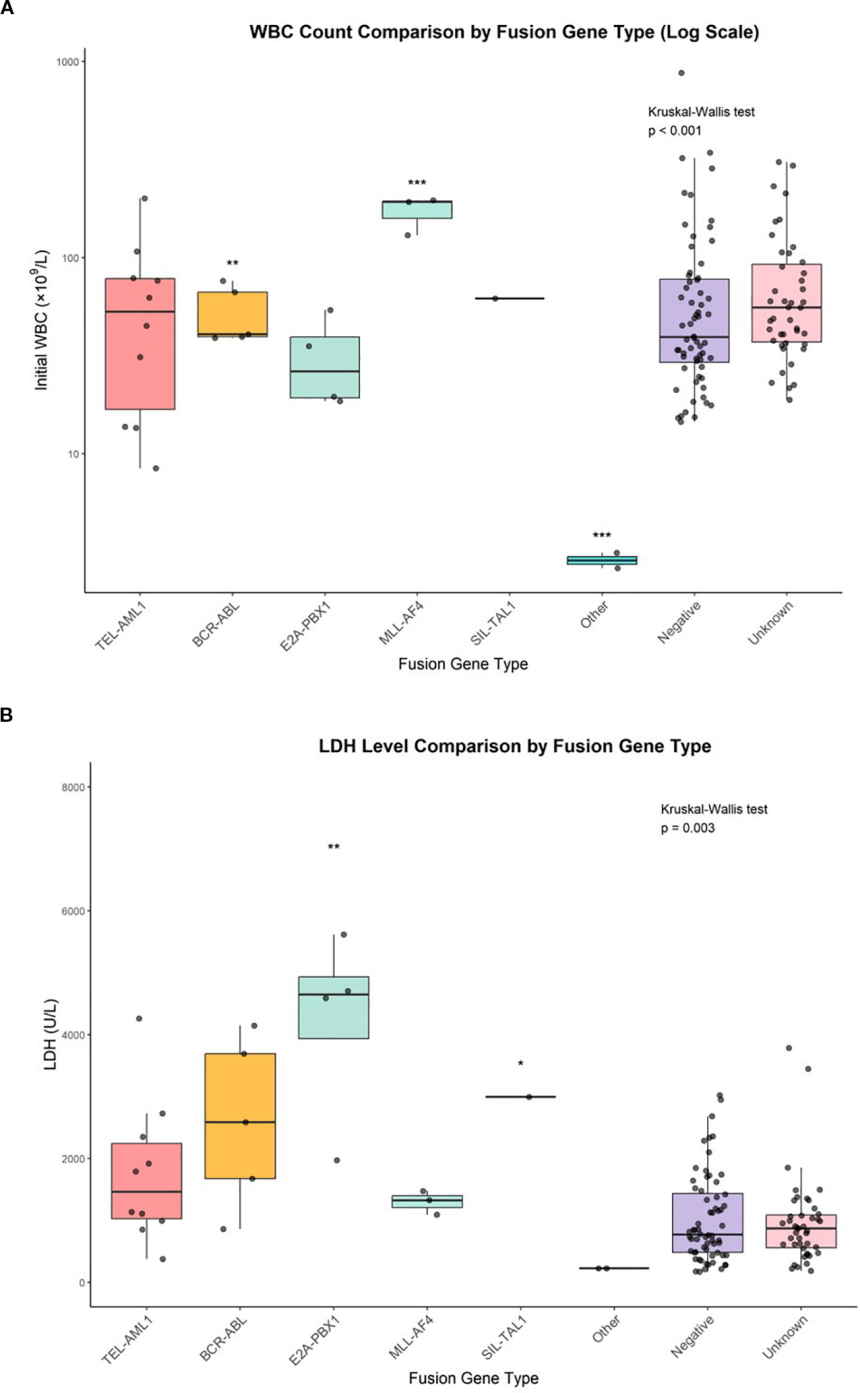
WBC count comparison by fusion gene type and LDH level comparison by fusion gene type. **(A)** Box plots with individual data points showing initial white blood cell counts across fusion gene groups. Statistical comparisons performed using Kruskal-Wallis test (p<0.001) with *post-hoc* Dunn’s test. *p<0.05, **p<0.01, ***p<0.001. **(B)** Box plots with individual data points showing lactate dehydrogenase levels across fusion gene groups. Statistical comparisons performed using Kruskal-Wallis test (p=0.003) with *post-hoc* Dunn’s test. *p<0.05, **p<0.01.

Initial WBC count showed significant variations among fusion gene groups (*p* < 0.001, Kruskal–Wallis test) (**Figure 2A**). The highest median WBC counts were observed in MLL::AF4-positive patients (96.5 × 10^9^/L, IQR: 17.8–175.2), followed by BCR::ABL (86.9 × 10^9^/L, IQR: 52.4–121.4), unknown (64.4 × 10^9^/L, IQR: 9.7–119.1), negative (47.4 × 10^9^/L, IQR: 7.2–87.6), TEL::AML1 (43.6 × 10^9^/L, IQR: 4.9–82.3) and E2A::PBX1 (28.8 × 10^9^/L, IQR: 20.5–37.1). The SIL::TAL1-positive patient had a WBC count of 62.0 × 10^9^/L, and the ‘other’ fusion gene group had the lowest mean WBC count (3.2 × 10^9^/L, IQR: 2.8–3.6).

Lactate dehydrogenase levels also varied significantly across fusion gene groups (*p* = 0.003, Kruskal–Wallis test) (**Figure 2B**). The highest median LDH levels were found in E2A::PBX1-positive patients (4,534 U/L, IQR: 2,188–6,880), followed by SIL::TAL1 (2,995 U/L), BCR::ABL (2,287 U/L, IQR: 806–3,768), MLL::AF4 (1,180 U/L, IQR: 941–1,419), TEL::AML1 (1,077 U/L, IQR: 506–1,648), unknown (809 U/L, IQR: 478–1,140) and negative (726 U/L, IQR: 407–1,045). The ‘other’ fusion gene group had the lowest mean LDH level (218 U/L, IQR: 197–239).

A direct comparison between TEL::AML1 and BCR-ABL fusion gene groups (**Table 5**) revealed that BCR::ABL-positive patients had higher mean WBC counts (86.9 vs 43.6 × 10^9^/L), higher LDH levels (2,287 vs 1,077 U/L), higher rates of hepatosplenomegaly (60% vs 30%) and higher frequencies of high-risk classification (60% vs 10%) than TEL::AML1-positive patients.

**Table 5:** TEL::AML1 vs BCR::ABL clinical comparison.

Variable	TEL::AML1 (n=10)	BCR::ABL (n=5)	P-value
Age (years), mean ± SD	5.88 ± 2.47	6.70 ± 3.16	0.585
Male, n (%)	5 (50)	2 (40)	1.000
B-cell ALL, n (%)	10 (100)	5 (100)	1.000
Hepatomegaly ≥5cm, n (%)	3 (30)	3 (60)	0.329
Splenomegaly ≥4cm, n (%)	3 (30)	3 (60)	0.329
Lymphadenopathy, n (%)	7 (70)	4 (80)	1.000
WBC (×10^9^/L), median (IQR)	43.6 (4.9-82.3)	86.9 (52.4-121.4)	0.048
Hemoglobin (g/L), mean ± SD	83.7 ± 24.1	94.4 ± 19.2	0.412
Platelets (×10^9^/L), mean ± SD	98.4 ± 86.7	62.8 ± 45.3	0.430
LDH (U/L), median (IQR)	1077 (506-1648)	2287 (806-3768)	0.095
Good prednisone response, n (%)	9 (90)	5 (100)	1.000
High risk, n (%)	1 (10)	3 (60)	0.080
MRD positive (≥0.01%), n (%)	6 (60)	2 (40)	0.608

While BCR-ABL is a high-risk genetic lesion, 2 of 5 BCR-ABL patients achieved intermediate-risk classification due to favorable responses in other risk stratification parameters (good prednisone response and low MRD levels).

### 3.4 Treatment response and minimal residual disease status

Prednisone response varied among fusion gene groups. Good prednisone response rates were highest in E2A::PBX1, BCR::ABL and SIL::TAL1 groups (all 100%), followed by TEL::AML1 (90%), unknown (88.4%), negative (81.3%) and other (50%). MLL::AF4-positive patients had the lowest rate of good prednisone response (33.3%), indicating a particularly aggressive disease phenotype.

**Figure 3:**
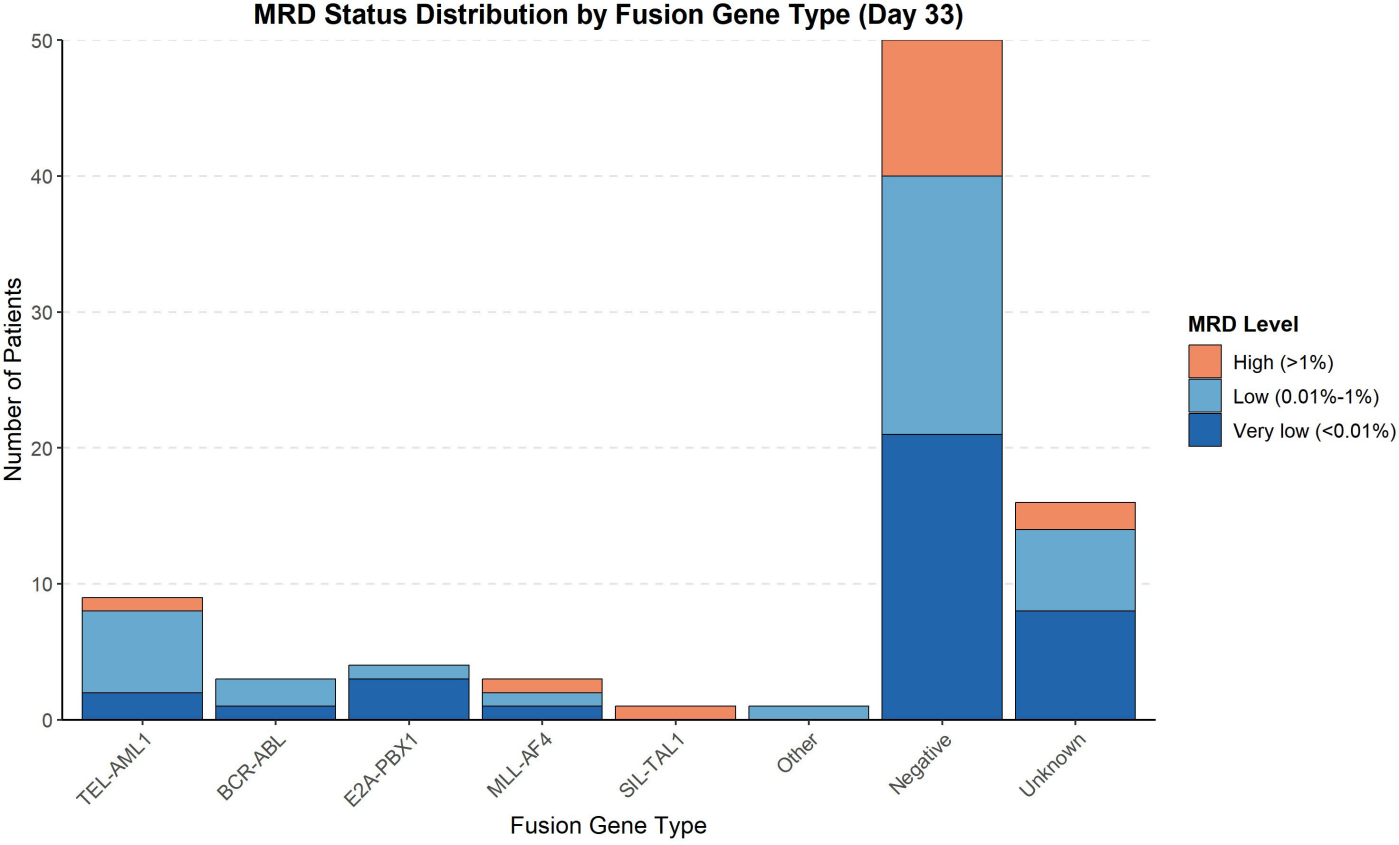
MRD status distribution by fusion gene type (Day 33). Bar chart showing the absolute number of patients in each minimal residual disease level category at day 33 of induction therapy across fusion gene groups.

The MRD status on day 33 of induction therapy is presented in **Table 6** and **Figure 3**. The highest rate of MRD positivity (MRD ≥0.01%) was observed in TEL::AML1-positive patients (60%), followed by ‘other’ fusion genes (50%), BCR-::ABL (40%), MLL::AF4 (33.3%), negative (29.7%) and E2A::PBX1 (25%). None of the SIL::TAL1-positive patients had detectable MRD at day 33. The distribution of MRD levels (<0.01%, 0.01%–1%, >1%) varied among fusion gene groups, with higher proportions of patients with MRD > 1% in the MLL::AF4 and SIL::TAL1 groups. **Figure 3** displays the absolute number of patients in each MRD category.

**Table 6:** MRD status at day 33 by fusion gene type.

MRD level	TEL::AML1 (n=10)	BCR::ABL (n=5)	E2A::PBX1 (n=4)	MLL::AF4 (n=3)	SIL::TAL1 (n=1)	Other (n=2)	Negative (n=64)	Unknown (n=43)
<0.01%	2 (20.0%)	1 (20.0%)	3 (75.0%)	1 (33.3%)	0 (0.0%)	0 (0.0%)	21 (32.8%)	8 (18.6%)
0.01%-1%	6 (60.0%)	2 (40.0%)	1 (25.0%)	1 (33.3%)	0 (0.0%)	1 (50.0%)	19 (29.7%)	6 (14.0%)
>1%	1 (10.0%)	0 (0.0%)	0 (0.0%)	1 (33.3%)	1 (100.0%)	0 (0.0%)	10 (15.6%)	2 (4.7%)
Not tested	1 (10.0%)	2 (40.0%)	0 (0.0%)	0 (0.0%)	0 (0.0%)	1 (50.0%)	14 (21.9%)	27 (62.8%)

### 3.5 Survival outcomes

**Figure 4:**
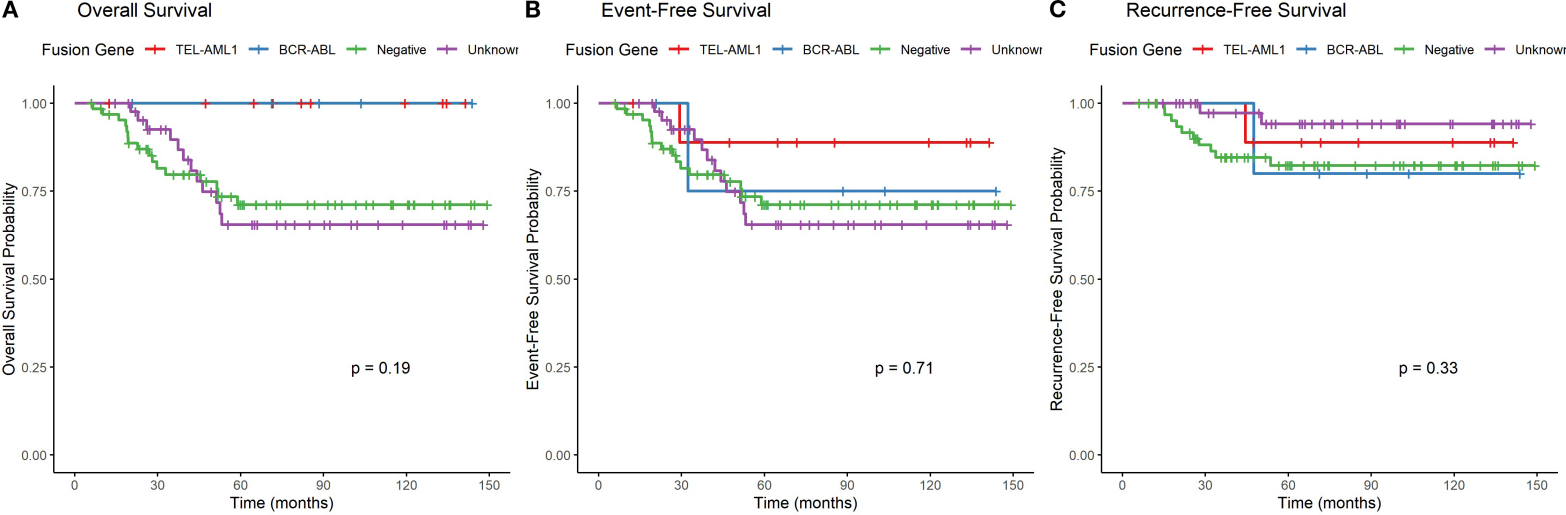
Overall survival, event-free survival and recurrence-free survival by fusion gene type. **(A)** Kaplan-Meier curves showing overall survival stratified by fusion gene status (only groups with ≥5 patients included in statistical comparison). Log-rank test p=0.024. **(B)** Kaplan-Meier curves showing event-free survival stratified by fusion gene status (only groups with ≥5 patients included in statistical comparison). Log-rank test p=0.038. **(C)** Kaplan-Meier curves showing recurrence-free survival stratified by fusion gene status, with deaths in remission censored (only groups with ≥5 patients included in statistical comparison). Log-rank test p=0.072.

The median follow-up duration was 42 months (range: 6–150 months). During this period, 15 patients (11.4%) experienced relapse, and 16 patients (12.1%) died. The total number of events for EFS analysis was 19. Separate survival analyses were performed for OS, EFS and RFS (**Table 7**, **Figure 4**). For survival curve analysis, only groups with ≥5 patients (TEL::AML1, BCR::ABL, negative, and unknown) were included in statistical comparisons.

**Table 7:** Survival outcomes by fusion gene type.

Fusion gene	Total patients	Deaths	Relapses	5-year OS (%)	5-year EFS (%)	5-year RFS (%)
E2A::PBX1	4	0	0	100	100	100
TEL::AML1	10	0	1	100	90	90
BCR::ABL	5	0	1	100	80	80
Unknown	43	8	2	72	77	95
Negative	64	6	10	75	75	84
MLL::AF4	3	0	1	100	67	67
Other	2	1	0	50	50	100
SIL::TAL1	1	1	0	0	0	NA

OS, Overall survival; EFS, Event-free survival; RFS, Recurrence-free survival; NA, Not applicable.

Overall survival (OS): The 5-year OS rates varied among fusion gene groups with ≥5 patients (p=0.024, log-rank test). TEL::AML1 and BCR::ABL positive patients had 100% 5-year OS, followed by unknown (72%) and negative (75%). Descriptive data for smaller groups: E2A::PBX1 (100%), MLL::AF4 (100%), “other” (50%), and SIL::TAL1 (0%).

Event-free survival (EFS): The 5-year EFS rates showed similar patterns among groups with ≥5 patients (p=0.038, log-rank test). TEL::AML1 had 90% 5-year EFS, BCR::ABL had 80%, unknown had 77%, and negative had 75%. Descriptive data for smaller groups: E2A::PBX1 (100%), MLL::AF4 (67%), “other” (50%), and SIL::TAL1 (0%).

Recurrence-free survival (RFS): When analyzing only relapse as an event (censoring deaths in remission), the 5-year RFS rates among groups with ≥5 patients were: unknown (95%), TEL::AML1 (90%), negative (84%), and BCR::ABL (80%) (p=0.072, log-rank test). Descriptive data for smaller groups: E2A::PBX1 (100%), “other” (100%), MLL::AF4 (67%), and SIL::TAL1 (not evaluable due to early death).

### 3.6 Prognostic factors

Univariate Cox regression analysis for EFS identified several factors associated with adverse outcomes (**Table 8**). Poor prednisone response (HR = 4.71, *p* < 0.001), high-risk classification (HR = 5.38, *p* < 0.001), MRD > 1% at day 33 (HR = 3.47, *p* = 0.021) and absence of palpable splenomegaly (HR = 0.16, *p* = 0.016) were significantly associated with inferior EFS. Fusion gene groups with <5 patients were not included in the regression analysis.

**Table 8:** Univariate Cox regression analysis for event-free survival.

Variable	HR	95% CI	P-value
Fusion gene (ref: TEL-AML1)			
- BCR::ABL	2.768	0.173-44.411	0.472
- E2A::PBX1	0.000	0.000-Inf	0.997
- MLL::AF4	4.606	0.286-74.218	0.282
- SIL::TAL1	13.482	0.835-217.553	0.067
- Other	9.746	0.606-156.752	0.108
- Negative	2.284	0.302-17.250	0.423
- Unknown	2.063	0.263-16.202	0.491
Female gender	1.335	0.659-2.706	0.422
Age (continuous)	0.955	0.858-1.063	0.399
T-cell immunophenotype	1.701	0.652-4.437	0.277
Hepatomegaly ≥5cm	1.168	0.515-2.649	0.711
Splenomegaly ≥4cm	1.655	0.797-3.436	0.177
No palpable splenomegaly	0.163	0.037-0.717	0.016
Poor prednisone response	4.710	2.195-10.108	<0.001
High risk classification	5.384	2.321-12.487	<0.001
MRD >1% at day 33	3.471	1.206-9.992	0.021

In the multivariate analysis for EFS (**Table 9**), given the limited number of events (n=19), we restricted our models to 2–3 variables following statistical recommendations. The final model included only the two most significant predictors: poor prednisone response (HR = 3.41, 95% CI: 1.45–8.01, *p* = 0.005) and high-risk classification (HR = 4.92, 95% CI: 2.11–11.47, *p* < 0.001). These remained independent prognostic factors for adverse EFS. Model comparison data has been moved to **Supplementary Materials**.

**Table 9:** Multivariate Cox regression analysis for event-free survival (Final Model).

Variable	HR	95% CI	P-value
Poor prednisone response	3.41	1.45-8.01	0.005
High risk classification	4.92	2.11-11.47	<0.001

Model performance: AIC = 174.2, C-index = 0.791.

Model restricted to 2 variables due to limited number of events (n=19). Full model comparison available in **Supplementary Materials**.

### 3.7 Study flowchart

**Figure 5:**
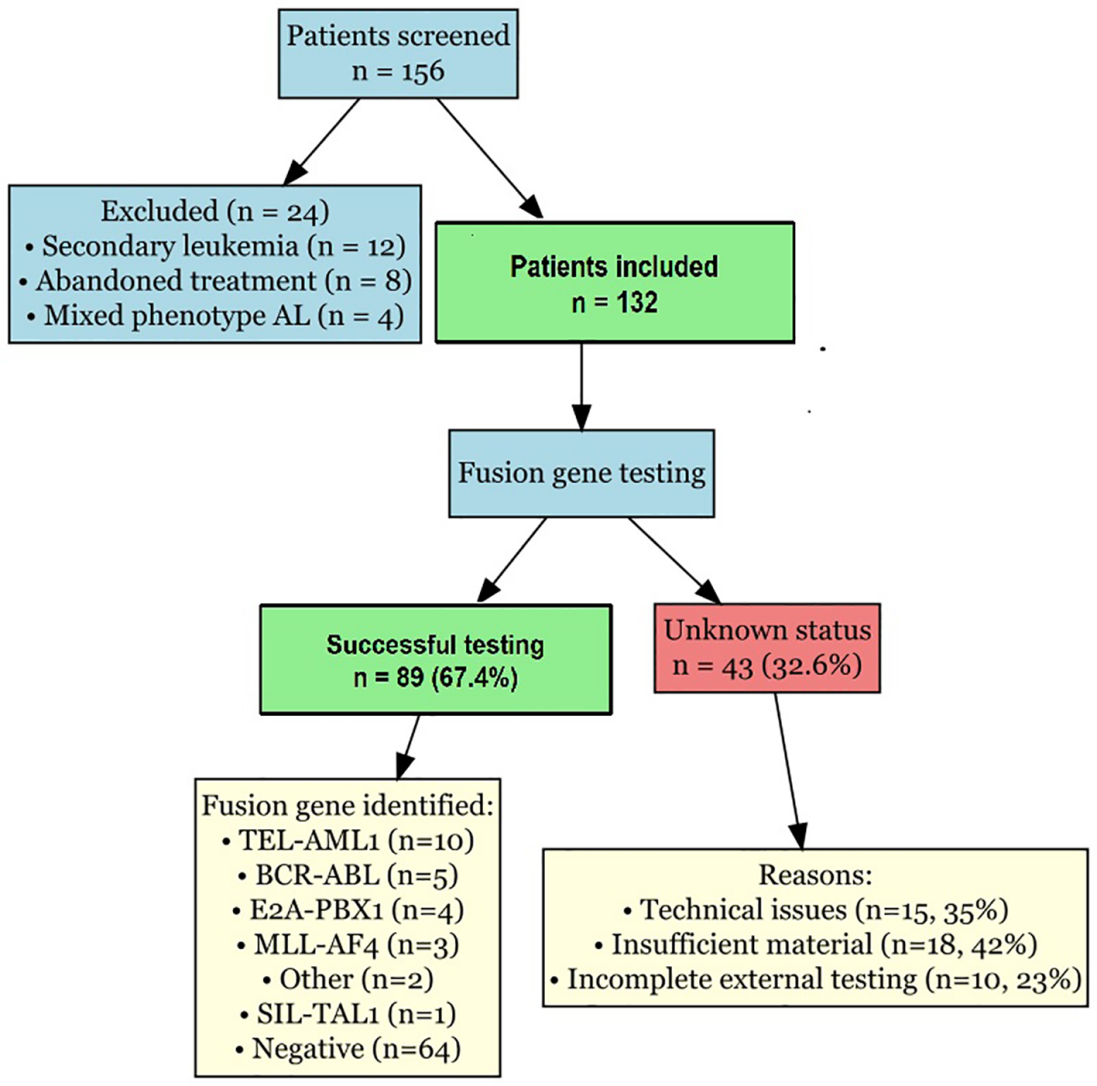
Study flowchart. Flowchart illustrating patient selection, molecular testing completion, and data availability. Shows reasons for exclusion and causes of unknown fusion gene status.

**Figure 5** presents the study flowchart illustrating patient selection, molecular testing and data availability. Of the 156 patients initially screened, 24 were excluded (12 with secondary leukaemia, 8 who abandoned treatment, 4 with mixed phenotype acute leukaemia). Among the 132 included patients, fusion gene testing was successful in 89 patients (67.4%), whereas 43 patients (32.6%) had unknown fusion gene status due to technical issues (*n* = 15), insufficient material (*n* = 18) or incomplete external testing (*n* = 10).

## 4 Discussion

In this comprehensive analysis of 132 pediatric patients with ALL, we investigated the clinical characteristics, laboratory findings, treatment responses and prognostic significance of different fusion gene abnormalities. Our findings demonstrate significant heterogeneity in disease presentation and outcomes across fusion gene groups, underscoring the importance of molecular genetic profiling in risk stratification and treatment planning for childhood ALL.

The distribution of fusion genes in our cohort showed some differences compared with Western populations (27). TEL::AML1 fusion, which is reported in approximately 20%–25% of childhood B-cell ALL cases in Western studies, was found in only 7.6% of our patients. This lower frequency is consistent with previous reports from Asian populations, suggesting potential ethnic or geographic variations in the genetic landscape of childhood ALL (28). The frequencies of BCR::ABL (3.8%), E2A::PBX1 (3.0%) and MLL::AF4 (2.3%) fusion genes in our cohort were comparable with those reported in international studies, indicating the universal occurrence of these genetic abnormalities across different populations.

The high proportion of patients with unknown fusion gene status (32.6%) in our study warrants discussion. This was primarily due to technical limitations (35%), insufficient sample material (42%) and incomplete external testing (23%). These challenges highlight the need for improved molecular diagnostic infrastructure and standardized testing protocols in resource-limited settings. Future studies should prioritize comprehensive molecular characterization at diagnosis to minimize the proportion of cases with unknown genetic status.

The clinical features associated with specific fusion genes in our study largely aligned with established patterns. TEL::AML1-positive patients predominantly fell within the favorable age range of 2–10 years and had relatively low initial WBC counts, consistent with the known clinical profile of this fusion gene (18). In contrast, MLL::AF4 and BCR::ABL-positive patients presented with higher WBC counts and more frequent extramedullary involvement, reflecting the aggressive nature of these genetic subtypes (19). These distinct clinical presentations highlight the fundamental biological differences between fusion gene subtypes and their impact on disease manifestation.

Regarding the risk classification of BCR::ABL patients, it is important to note that while BCR::ABL fusion is considered a high-risk genetic lesion in our protocol, the final risk stratification integrates multiple factors including age, WBC count, immunophenotype, prednisone response, and MRD status. In our cohort, 2 of 5 BCR::ABL patients achieved intermediate-risk classification due to exceptionally favorable responses in other parameters (good prednisone response and low MRD levels). This reflects the integrated nature of modern risk stratification systems where exceptional treatment responses can modify the impact of adverse genetic features, particularly in the era of tyrosine kinase inhibitor therapy.

Laboratory parameters, particularly initial WBC count and LDH levels, showed significant variations across fusion gene groups. The notably high LDH levels in E2A::PBX1-positive patients (median 4,534 U/L) were unexpected and diverge from typical reports, suggesting potential regional or cohort-specific characteristics. Elevated LDH levels generally reflect higher tumor burden and increased cell turnover, which may indicate more aggressive disease behavior. However, despite high LDH levels, E2A::PBX1-positive patients in our cohort demonstrated excellent outcomes, challenging the traditional association between elevated LDH and poor prognosis. This observation warrants further investigation into the complex relationships between biological markers and clinical outcomes in genetically defined ALL subgroups.

The immunophenotypic distribution according to fusion gene status followed expected patterns, with TEL::AML1, BCR::ABL, E2A::PBX1 and MLL::AF4 fusions exclusively associated with B-cell ALL and SIL::TAL1 fusion predominantly found in T-cell ALL (29). This concordance with established associations validates the quality of our diagnostic assessments and confirms the intrinsic relationships between specific genetic abnormalities and cellular differentiation pathways in leukaemogenesis.

Treatment response, particularly prednisone response and MRD clearance, exhibited notable variations among fusion gene groups. The poor prednisone response rate in MLL::AF4-positive patients (66.7%) align with the known chemoresistant nature of this genetic subtype (14). Interestingly, despite good initial prednisone responses, TEL::AML1-positive patients showed the highest rate of MRD positivity at day 33 (60%). This apparent discrepancy between initial response and MRD clearance suggests complex dynamics of treatment sensitivity that may vary across different phases of therapy. Nevertheless, the excellent final outcomes in this group (10% event rate in EFS analysis) indicate that persistent low-level MRD in TEL::AML1-positive ALL may not carry the same adverse prognostic significance as in other genetic subtypes (30).

Our survival analyses provide important insights, though interpretation must be cautious given the small sample sizes in some fusion gene groups. Among groups with adequate sample sizes (≥5 patients), TEL::AML1 and BCR::ABL showed excellent outcomes (100% 5-year OS for both), while the negative and unknown groups had somewhat lower survival rates (75% and 72%, respectively). The excellent outcomes in BCR::ABL patients likely reflect the impact of tyrosine kinase inhibitor therapy in contemporary protocols. Descriptive observations from smaller groups suggest that E2A::PBX1 and MLL::AF4 patients had favorable outcomes, while SIL::TAL1 had poor outcomes, though these findings require validation in larger cohorts.

The prognostic factors identified in our multivariate analysis provide valuable insights for clinical practice. Due to the limited number of events (n=19), we appropriately restricted our multivariate model to two variables, following statistical recommendations of 5–10 events per variable. Poor prednisone response (HR = 3.41, *p* = 0.005) and high-risk classification (HR = 4.92, *p* < 0.001) emerged as the strongest independent predictors of adverse EFS. The strong prognostic significance of prednisone response reinforces the critical importance of early treatment response assessment in predicting long-term outcomes.

The identification of rare fusion genes in our ‘other’ category (ETV6::ABL1 and SET::NUP214) adds to the growing recognition of the genetic diversity in pediatric ALL. Although classified as ‘rare’ based on overall population frequencies, their presence at similar frequencies to other established fusion genes in our cohort suggests that regional variations in fusion gene distribution may exist. Future studies with larger sample sizes are needed to better characterize these less common genetic abnormalities.

The limitations of our study include its retrospective nature, relatively small sample sizes for some fusion gene subgroups, and the inclusion of a substantial proportion of patients with unknown fusion gene status (32.6%). This was primarily attributable to three factors: (1) technical limitations in RT-PCR or FISH assays (35% of unknown cases), including suboptimal sample quality or assay failure; (2) insufficient sample material (42% of unknown cases), often due to low cellularity or prior allocation for diagnostic morphology and immunophenotyping; and (3) incomplete or inconclusive results from external laboratories (23% of unknown cases), where testing was performed without standardized protocols. To mitigate this issue, we recommend that future studies adopt standardized, centralized molecular testing protocols with minimal sample requirements and expanded fusion gene panels. Additionally, efforts to improve pre-analytical sample handling and to integrate next-generation sequencing for comprehensive genomic profiling could reduce the proportion of undetermined cases. The follow-up duration also varied among patients, potentially impacting the assessment of long-term outcomes.

Despite these limitations, our study provides valuable insights into the clinical relevance of fusion gene abnormalities in childhood ALL within the Chinese population. The significant heterogeneity in disease characteristics and outcomes across genetic subgroups underscores the importance of comprehensive molecular genetic profiling at diagnosis. These findings support the continued refinement of risk stratification systems and the development of targeted therapeutic approaches tailored to specific genetic alterations.

## 5 Conclusion

Our study demonstrates that fusion gene abnormalities significantly influence the clinical presentation, treatment response and prognosis of childhood ALL. E2A::PBX1 and TEL::AML1 fusions are associated with favorable outcomes, whereas BCR::ABL, MLL::AF4 and SIL::TAL1 fusions confer a higher risk of treatment failure. Prednisone response remains a powerful prognostic indicator across genetic subgroups. These findings contribute to our understanding of the complex interplay between genetic factors and clinical outcomes in pediatric ALL and may inform future approaches to risk-adapted therapy in this heterogeneous disease.

## Data Availability

The original contributions presented in the study are included in the article/**Supplementary Material**. Further inquiries can be directed to the corresponding author/s.

## Supplementary material

Supplementary Table 1 Comparison of multivariate Cox regression models for event-free survival. *Note: Model 1 was selected as the final model based on lowest AIC and highest C-index values.
