# Supplementary material for: Clinical characteristics and prognostic analysis of different fusion gene abnormalities in childhood acute lymphoblastic leukaemia
Source: Front Oncol. 2025 Oct 29;15:1616686. doi: 10.3389/fonc.2025.1616686 (PMC12605346; doi:10.3389/fonc.2025.1616686)
Supplement: Supplementary Table 1 — Comparison of multivariate Cox regression models for event-free survival. *Note: Model 1 was selected as the final model based on lowest AIC and highest C-index values. [file Table1.docx]

**Supplementary Table S1. Comparison of multivariate Cox regression models for event-free survival**

| **Model** | **Variables** | **AIC** | **C-index** |
| --- | --- | --- | --- |
| Model 1 | Prednisone response + Risk classification | 174.2 | 0.791 |
| Model 2 | Prednisone response + MRD >1% | 176.8 | 0.773 |
| Model 3 | Risk classification + Splenomegaly | 175.4 | 0.782 |

*Note: Model 1 was selected as the final model based on lowest AIC and highest C-index values.
